# Genome Sequence of the Deltaproteobacterial Strain NaphS2 and Analysis of Differential Gene Expression during Anaerobic Growth on Naphthalene

**DOI:** 10.1371/journal.pone.0014072

**Published:** 2010-11-19

**Authors:** Raymond J. DiDonato, Nelson D. Young, Jessica E. Butler, Kuk-Jeong Chin, Kim K. Hixson, Paula Mouser, Mary S. Lipton, Robert DeBoy, Barbara A. Methé

**Affiliations:** 1 Microbiology Department, University of Massachusetts, Amherst, Massachusetts, United States of America; 2 Department of Biology, Georgia State University, Atlanta, Georgia, United States of America; 3 Biological Sciences Division, Pacific Northwest National Laboratory, Richland, Washington, United States of America; 4 J. Craig Venter Institute, Rockville, Maryland, United States of America; Cinvestav, Mexico

## Abstract

**Background:**

Anaerobic polycyclic hydrocarbon (PAH) degradation coupled to sulfate reduction may be an important mechanism for in situ remediation of contaminated sediments. Steps involved in the anaerobic degradation of 2-methylnaphthalene have been described in the sulfate reducing strains NaphS3, NaphS6 and N47. Evidence from N47 suggests that naphthalene degradation involves 2-methylnaphthalene as an intermediate, whereas evidence in NaphS2, NaphS3 and NaphS6 suggests a mechanism for naphthalene degradation that does not involve 2-methylnaphthalene. To further characterize pathways involved in naphthalene degradation in NaphS2, the draft genome was sequenced, and gene and protein expression examined.

**Results:**

Draft genome sequencing, gene expression analysis, and proteomic analysis revealed that NaphS2 degrades naphthoyl-CoA in a manner analogous to benzoyl-CoA degradation. Genes including the previously characterized NmsA, thought to encode an enzyme necessary for 2-methylnaphthalene metabolism, were not upregulated during growth of NaphS2 on naphthalene, nor were the corresponding protein products. NaphS2 may possess a non-classical dearomatizing enzyme for benzoate degradation, similar to one previously characterized in *Geobacter metallireducens*. Identification of genes involved in toluene degradation in NaphS2 led us to determine that NaphS2 degrades toluene, a previously unreported capacity. The genome sequence also suggests that NaphS2 may degrade other monoaromatic compounds.

**Conclusion:**

This study demonstrates that steps leading to the degradation of 2-naphthoyl-CoA are conserved between NaphS2 and N47, however while NaphS2 possesses the capacity to degrade 2-methylnaphthalene, naphthalene degradation likely does not proceed via 2-methylnaphthalene. Instead, carboxylation or another form of activation may serve as the first step in naphthalene degradation. Degradation of toluene and 2-methylnaphthalene, and the presence of at least one *bss*-like and *bbs*-like gene cluster in this organism, suggests that NaphS2 degrades both compounds via parallel mechanisms. Elucidation of the key genes necessary for anaerobic naphthalene degradation may provide the ability to track naphthalene degradation through in situ transcript monitoring.

## Introduction

Anaerobic microbial oxidation of polycyclic aromatic hydrocarbons (PAHs) coupled to the reduction of sulfate can be an important mechanism for the removal of PAHs from contaminated marine sediments [1], [2]. When the natural process of PAH-contaminant removal is sufficiently active it may alleviate the need for more invasive and expensive remediation strategies such as dredging to remove the sediments [3]. However, documenting the activity of anaerobic PAH-degrading microorganisms in contaminated marine sediments can be problematic For example, anaerobic oxidation of PAHs in sediments can be documented as the production of ^14^CO_2_ from [^14^C]-labeled PAHs [1], [4], [5], [6]. However, subsequent studies demonstrated that, depending upon the PAH of interest, mineralization of [^14^C]-PAHs can either underestimate or overestimate the rate of degradation of the *in situ* PAH pools. It is possible to estimate rates of PAH degradation in long-term sediment incubations in which the loss of the PAHs from the sediments is documented over time[2]. Not only does this approach require a high level of analytical precision, but it may take years to accurately determine whether PAHs are being degraded. Therefore, this approach is unlikely to ever be routinely adopted.

An alternative strategy is to document whether PAH-degrading sulfate-reducing microorganisms are expressing the genes and/or enzymes involved in PAH degradation. Recent studies have demonstrated that the abundance of transcripts for key metabolic genes can be related to the rates of microbial metabolism in soils and sediments [7]–[12]. For example, quantification of in situ expression levels of dissimilatory (bi)sulfite reductase (*dsr*) was used to track the rate of sulfate reduction in petroleum-contaminated marine sediments, and transcript levels of the *dsrA* gene correlated well with sulfate reduction rates[10]. Likewise, expression levels of citrate synthase have been used to track rates of central metabolism in *Geobacter.* Citrate sythase expression levels increase with growth rates whereas expression rates for housekeeping genes do not [11]. In a study of methanogenesis, expression rates (in this case transcript/gene ratios for *mcrA*) were found to increase linearly with the methanogenesis process [12].

Studies have suggested that the first step of naphthalene degradation is either a carboxylation or a methylation [13], [14]. Carboxylation of naphthalene was demonstrated using [^13^C]labeled bicarbonate cultures enriched from sediments isolated in the Arthur Kill, and is supported by growth of NaphS2 on 2-naphthoate [15]. Methylation as the initial step for naphthalene degradation was demonstrated by radiolabeling studies in which enrichment culture N47 was fed either deuterated naphthalene or [^13^C]labeled bicarbonate, and the authors concluded that, although the initial characterization of this culture had suggested the first step of naphthalene degradation was a carboxylation [14], it had not been possible to distinguish between a methylation or carboxylation in this study [16], [17], [18]. Though growth on 2-naphthoate suggests that this is a likely product in the naphthalene degradation pathway of NaphS2, it is unclear which mode, whether carboxylation or methylation, occurs in NaphS2. In the enrichment culture N47, the isolated enzymatic activities of succinyl-CoA:naphthyl-2-methyl-succinate CoA transferase and naphthyl-2-methyl-succinyl-CoA dehydrogenase were consistent with previous radiolabeling studies and suggested that steps analogous to benzylsuccinate synthase pathway in the toluene degradation pathway activates naphthalene via a fumarate addition and subsequent conversion to 2-naphthoic acid CoA [16], [19]. It has been suggested that later steps in naphthalene degradation occur in a pathway analogous to the benzoyl-CoA reductase pathway [13], [16], although degradation products formed from tetralin degradation suggested that these steps would involve cyclohexanoic compounds rather than monoaromatic intermediates as in benzoate degradation [16].

Aspects of the pathway for anaerobic 2-methylnaphthalene degradation by sulfate reducers have recently been elucidated [20], [21]. In NaphS2, NaphS3, and NaphS6, the *nmsA* gene product, encoding the alpha subunit of naphthyl 2-methylsuccinate synthase, which is proposed to convert 2-methylnaphthalene to naphthyl 2-methylsuccinate, was identified as induced during growth on 2-methylnaphthalene, but not during growth on naphthalene. Moreover, while the strains NaphS2, NaphS3, and NaphS6 metabolize both naphthalene and 2-methylnaphthalene, a lag in growth upon transfer of these cultures from naphthalene to 2-methylnaphthalene as the primary electron donor suggests that enzymes necessary for 2-methylnaphthalene degradation are not active during growth on naphthalene, and that 2-methylnaphthalene is not an intermediate during naphthalene degradation in these species. Growth of the enriched culture N47 on 2-methylnaphthalene has further elucidated the 2-methylnaphthalene degradation pathway. Genome sequencing and proteomic analysis of N47 revealed that *nmsABC*, which encode subunits of naphthyl 2-methylsuccinate synthase and are similar to the toluene degradation genes *bssABC*, are expressed during growth on 2-methylnaphthalene. Genes encoding *bnsA-H* (*b*eta oxidation of *n*aphthyl 2-methyl*s*uccinate), which are thought to be involved in conversion of naphthyl 2-methylsuccinate to naphthoyl-CoA, were also isolated in N47 during growth on 2-methylnaphthalene. These genes are similar to the *bbs* genes of *T. aromatica*, *Azoarcus* species, and *Aromatoleum aromaticum* strain EbN1, which are also involved in anaerobic toluene degradation. Also in N47, a cluster of genes, induced during growth on 2-methylnaphthalene, has been identified as encoding subunits of naphthoyl-CoA reductase, suggesting a mechanism of degradation of 2-naphthoyl-CoA that is analogous to benzoyl-CoA reductase degradation. These genes encode proteins (NcrABCD) with similarity to BcrABCD in *A. aromaticum* EbN1. Interestingly, genes encoding homologs of BamB-I, which encode components postulated to be involved in benzoyl-CoA dearomatization in *Geobacter metallireducens*, were not present in the genome of N47 [22], [23].

In order to further elucidate the genetic and biochemical mechanisms underlying anaerobic naphthalene degradation, we sequenced the genome of the naphthalene degrading, marine sulfate reducer NaphS2. Microarray analysis of whole genome expression, as well as proteomics analysis, was conducted for NaphS2 cultures grown on benzoate compared to cultures grown on pyruvate and also for cultures grown on naphthalene compared to cultures grown on benzoate. These studies revealed gene clusters involved in naphthalene degradation, and suggested that *nmsABC* and *bnsA-H* genes, while present in NaphS2 and previously implicated in 2-methylnaphthalene degradation, are not expressed during growth on naphthalene. However, enzymes similar to benzoyl-CoA reductase are induced during naphthalene degradation. Our results suggest that while NaphS2 has the capacity to degrade 2-methylnaphthalene, consistent with previous studies and the presence of *nmsABC* and *bnsA-H* in the NaphS2 genome, the pathway for 2-methylnaphthalene degradation does not appear to be active during NaphS2 growth on naphthalene. Together, these results suggest that 2-methylnaphthalene likely is not an intermediate of naphthalene degradation in NaphS2, though it is unclear how naphthalene is initially activated for degradation. We also show that NaphS2 is capable of degrading toluene, suggesting that the mechanisms for 2-methylnaphthalene and toluene degradation may be catalyzed by the same or similar suites of enzymes in NaphS2, given the presence of at least one *bss-* and *bbs*-like gene cluster in the draft NaphS2 genome. Future experiments should examine whether *nmsABC* and *bnsA-H* are induced during growth on toluene and 2-methylnaphthalene in NaphS2.

## Results

### Sequencing

Whole genome shotgun sequencing of the NaphS2 genome was performed to produce a draft genome represented by 188 scaffolds with a total estimated genome size of 6,586,693 base pairs and 7329 predicted Open Reading Frames (ORFSs). Annotation of ORFS assigned 6398 (87%) to a function. Results of best taxonomic matches to the set of predicted ORFs from Naph2 using Blast confirmed that the majority of these matches are to members of the ∂-proteobacteria, thus placing this genome within the δ-proteobacteria lineage.

### Naphthalene Degradation

In order to elucidate the pathway for naphthalene degradation in NaphS2, DNA microarrays consisting of oligonucleotide sequences corresponding to the predicted ORFs from the draft NaphS2 genome were hybridized with linearly amplified RNA from cultures grown with naphthalene, benzoate, or pyruvate as the electron donor and carbon source and with sulfate as the electron acceptor. Comparisons were made of benzoate- versus pyruvate-grown mid-log batch cultures and naphthalene-versus benzoate-grown mid-log batch cultures. Proteomic analysis using LS-MS was used to determine relative protein levels under the same growth conditions.

**Figure 1:**
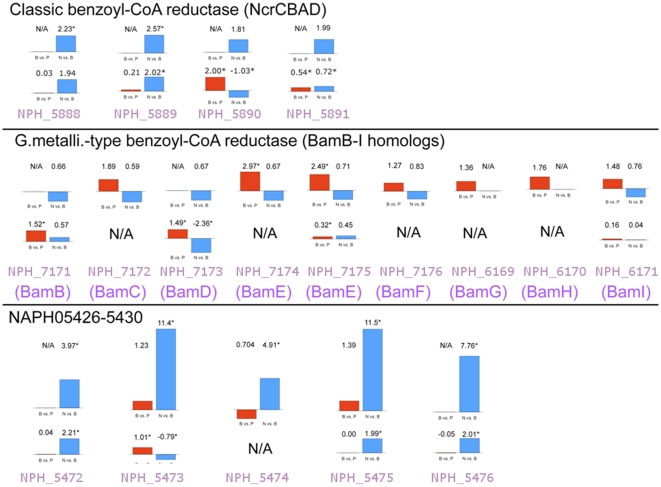
NaphS2 has three benzoyl-CoA reductases, with differing patterns of expression. Two panels are depicted for each gene displayed. Red bars represent mRNA or protein up/downregulation on benzoate vs. pyruvate, blue bars represent mRNA or protein up/downregulation on naphthalene vs. benzoate. For each set of genes, the upper panel shows mRNA up/down-regulation, benzoate vs. pyruvate, naphthalene vs. benzoate, in fold change units. Stars indicate significant changes (values >2). N/A indicates that no data was available. The lower panel shows protein up/down-regulation, with the same conventions used for depicting mRNA expression except that units are Z-score difference and significance was determined by the t-test.

In order to isolate genes involved in degradation of naphthalene but not benzoate, we examined gene expression during growth on naphthalene versus growth on benzoate. Close to 200 genes were differentially expressed during growth on naphthalene versus growth on benzoate (Table S1). A cluster of genes homologous to *Azoarcus bzdNOPQ* (Table S2), which encode benzoyl-CoA reductase, was upregulated during growth on naphthalene. Moreover, this gene cluster was not upregulated during growth on benzoate versus pyruvate, suggesting that it is not involved in benzoate degradation in NaphS2 (Figure 1, Tables S3, S4, S5). Given their upregulation specifically during growth on naphthalene, and given their similarity to genes isolated from N47 during growth on naphthalene [21], we postulate that this gene cluster encodes a naphthoyl-CoA reductase, and designated it *ncrABCD* (*n*aphthoyl-*C*oA *r*eductase) after nomenclature assigned to the N47 homologs. None of the genes upregulated during growth on naphthalene vs benzoate were likely candidates for the naphthyl-2-methyl-succinate synthase, succinyl-CoA:naphthyl-2-methyl-succinate CoA-transferase and naphthyl-2-methyl-succinyl-CoA dehydrogenase activities detected in enrichment culture N47, and homologs to *nmsABC* and *bnsA-H*, involved in degradation of 2-methylnaphthalene in N47 [18], [21], were not significantly upregulated in NaphS2 during growth on naphthalene, though *bnsG* showed an induction of 1.6 fold. Interestingly, comparing differential expression of growth on naphthalene to growth on pyruvate, *bnsG* and *bnsH* were differentially expressed by more than 2-fold, and *bnsA* was differentially expressed by just under 2-fold. Other genes thought to play a role in degradation of 2-methylnaphthalene in N47, *bnsE*, *bnsF*, and *nmsA*, the only gene identified in the NaphS2 draft genome encoding NmsA activity [20], showed expression ratios well below the 2-fold during growth on naphthalene vs pyruvate and no differential expression on naphthalene vs benzoate.

**Figure 2:**
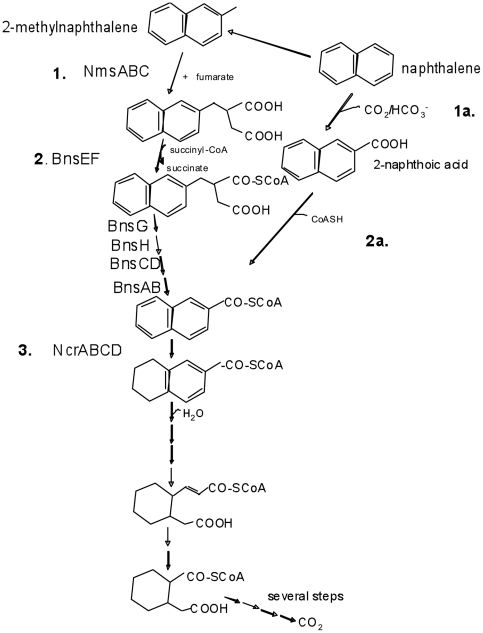
Proposed Pathways for Naphthalene and 2-Methylnaphthalene degradation. Steps 1–3 outline proposed steps involved in degradation of 2-methylnaphthalene. One model for naphthalene degradation involves the methylation of naphthalene to 2-methylnaphthalene and its subsequent degradation by Nms and Bns in steps analogous to toluene degradation. Steps 1a, 2a, and 3 outline a proposed mechanism for naphthalene degradation in which naphthalene is carboxylated and coadenylated. Later steps common to both the methylation and carboxylation models of naphthalene degradation include reduction of naphthoyl-CoA by NcrABCD (step 3).

We next identified candidate genes which might encode enzymes capable of coadenylating 2-naphthoic acid. A novel gene cluster (NPH_5472-77) was identified that is highly upregulated on naphthalene (Table S1) and contains a gene encoding a protein similar to phenylacetate-CoA ligases (NPH_5477; 50% similarity to *E. coli*, swissprot BLASTP). This gene cluster also encodes five oxidoreductase genes, NPH_5472-76, with similarity to a NADH dehydrogenase subunit E from *Rickettsia bellii* (NPH_5472; 35% identity, 58% similarity, swissprot BLASTP), a NADH-quinone oxidoreductase subunit F 2 from *Sinorhizobium meliloti* (NPH_5473; 45% identity, 67% similarity), a NADH-quinone oxidoreductase subunit G 2 from *Sinorhizobium meliloti* (NPH_5474; 37% identity, 50% similarity), a NADH oxidase from *Thermoanaerobacter brockii* (NPH_5475; 35% identity, 51% similarity) and a NADH-dependent flavin oxidoreductase from *Clostridium scindens* (NPH_5476; 31% identity, 50% similarity). These reductases may play a role in the initial ring dearomatization during naphthalene degradation, where NPH_5472-76 would catalyze the first ring dearomatization, whereas NcrABCD would catalyze the second ring reduction (Figure 2). Interestingly, three of these putative reductase genes (NPH_5472-4) have products homologous to BamG-BamI (65%, 71%, and 50% similarity respectively) of *G. metallireducens,* suggesting that these may act on naphthalene in a way analogous to how the Bam system acts during benzoate degradation, where it is postulated to dearomatize benzoyl-CoA.

**Figure 3:**
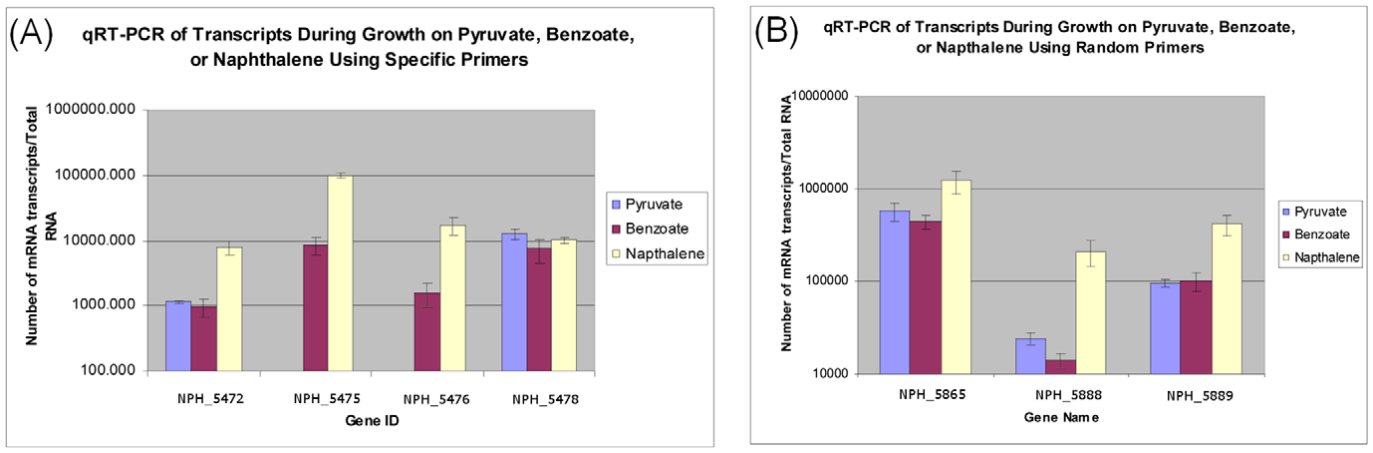
Quantitative RT-PCR analysis of transcripts during growth on pyruvate, benzoate, and naphthalene. Quantitative RT-PCR analysis was conducted to confirm expression of genes upregulated during growth on naphthalene, using RNA from microarray experiments for genes indicated in each panel. (A) Quantitative RT-PCR of select genes using gene specific primers during the reverse transcription reaction. (B) Quantitative RT-PCR of select genes using random primers during the reverse transcription reaction.

Real-time RT-PCR analysis was conducted to confirm expression of genes upregulated during growth on naphthalene. Expression trends for several genes mirrored the results of the microarray analyses. *NPH_5888* and *NPH_5889*, encoding the NcrC and NcrD subunits of naphthoyl-CoA reductase, were both upregulated during growth on naphthalene versus growth on either pyruvate or benzoate (Figure 3), confirming microarray results showing specific induction of genes encoding benzoyl-CoA reductase during growth on naphthalene. *NPH_5865*, *NPH_5472*, *NPH_5475*, and *NPH_5476* were also upregulated during growth on naphthalene, while a nearby gene, *NPH_5478*, was not differentially expressed (Figure 3), further confirming expression trends observed in microarray analyses and suggesting that *NPH_5472*-*NPH_5476* are co-regulated and may play a role in naphthalene degradation.

In order to examine proteins differentially expressed during benzoate and naphthalene degradation in NaphS2, FTICR-MS analysis was conducted on the soluble and insoluble fractions of three biological replicates analyzed in duplicate for each condition (naphthalene, benzoate, and pyruvate). Proteins displaying significant differences in expression between either benzoate versus pyruvate, naphthalene versus pyruvate, or naphthalene versus benzoate were identified using a paired t-test (p-value cut-off 0.01), and the average Z-scores for these statistically significant proteins were calculated for each condition (Tables S4-S5).

During growth on naphthalene versus growth on benzoate, NPH_5475 and NPH_5476, which are predicted oxidoreductases, and NPH_5477, a predicted phenylacetate-CoA ligase, were upregulated in the insoluble fraction, further confirming that a Bam-type system may be involved in naphthalene degradation. Several subunits of 4-hydroxybenzoate-CoA reductase (NPH_1040, NPH_1743, NPH_2863) and two predicted carboxylases with similarity to 3-octaprenyl-4-hydroxybenzoate carboxy-lyases (NPH_5859, NPH_5865), were also upregulated on naphthalene. NcrB and NcrD were also upregulated during growth on naphthalene, as were NPH_5899, and NPH_5901-NPH_5902. BzdT and NPH_3733 were upregulated on benzoate, as was BzdX and a cyclohexa-1,5-dienecarbonyl-CoA hydratase (NPH_5406). These proteins play a role in degradation steps subsequent to benzoyl-CoA reduction.

Though their respective transcripts were not significantly upregulated in microarray analysis, BnsD, a putative naphthyl-2-hydroxymethyl-succinyl-CoA dehydrogenase subunit, and BnsG, a putative naphthyl-2-methyl-succinyl-CoA dehydrogenase, were both upregulated on naphthalene, as was NPH_4694, a homolog to Gmet_1526, encoded by a gene adjacent to the *bns* gene cluster in NaphS2.

In the soluble naphthalene-grown fraction, NPH_5898, a 3-hydroxybutyryl-CoA dehydratase, NPH_5903, a conserved hypothetical protein, and NPH_5908, an acyl-CoA dehydrogenase were expressed. These proteins are encoded by genes downstream of the *ncr* gene cluster in NaphS2 and are likely involved in degradation steps subsequent to naphthoyl-CoA reduction.

### 2-Methylnaphthalene Degradation

Two clusters containing genes which encode homologs to toluene degradation enzymes were isolated from NaphS2, and one of them encodes NmsA, previously shown to be induced during growth on 2-methylnaphthalene degradation in the strains NaphS2, NaphS3, and NaphS6, as well as in N47 [20], [21]. This corresponding gene, *nmsA*, is present in a gene cluster containing homologs to N47 *nmsA* and *nmsC* (Table S2), which together with *nmsB*, encode naphthyl-2-methyl-succinate synthase, analogous to the BssABC complex involved in toluene degradation. The enzymatic steps in the conversion of toluene to benzyl-CoA are catalyzed by benzylsuccinate synthase (Bss) and benzylsuccinate coA transferase (Bbs) [24], [25]. Genes coding for the subunits of these enzymes appear to be present on separate contigs in the NaphS2 genome and in two distinct clusters. One contig contains *bbsA* followed by *bbsB*. A second contig contains the genes *bbsCDEFGH*, followed by orthologs to the *G. metallireducens* genes Gmet_1526, Gmet_1527, and Gmet_1528, which in the *G. metallireducens* genome are found in the toluene degradation cluster downstream of the *bbs* operon, and preceding *bssCAB*. Gmet_1526 and Gmet_1527 are similar to the alpha and beta subunits of a flavoprotein electron acceptor for benzylsuccinyl-CoA dehydrogenase [24], and Gmet_1528 encodes a hypothetical protein containing Fe-S oxidoreductase domain which is also postulated to be involved in electron transfer from benzylsuccinyl-CoA [24]. A third NaphS2 contig contains genes similar to *bbsDEFG* followed by orthologs to EbN1 genes c2B001, c2A200, c2A300, and c2A203, which are postulated to play a role in toluene degradation and which separate the *bss* and *bbs* gene clusters in EbN1[25]. Thus, the conservation of NaphS2 genes following the *bbs*-like gene clusters is more similar to that observed in *G. metallireducens*, whereas the *bss*-like gene cluster appears more similar to *A. aromatoleum* EbN1. Given that the *bssA*-like gene in NaphS2 is identical to *nmsA* previously characterized to be involved in 2-methylnaphthalene degradation, and given that there is only one *bss*-like and *bbs*-like cluster in NaphS2, we have designated the *bss*-like cluster as *nms*, and the *bbs*-like cluster *bns*, to be consistent with nomenclature used in N47 and since these genes are likely involved in 2-methylnaphthalene degradation in NaphS2.

The *nms* and *bns* gene products of NaphS2 show highest amino acid similarity to orthologs from *A. aromatoleum* EbN1and *G. metallireducens*, with the exception of BnsD, NmsD, and NmsC (Table S2). The sequence similarity of NmsD is relatively low compared to the similarities of other *bss* and *bns* gene products to their orthologs (Table S2). However, NaphS2 BnsD contains the conserved Cys motifs found in all BbsD proteins, consisting of one N-terminal C-x_3_-C-x_2_-C motif corresponding to a radical SAM domain and two C-x_2_-C-x_2_-C-x_3_-C motifs corresponding to ferredoxin domains [24]. The C-x_3_-C-x_2_-C motif is necessary for BssD activity, which involves activation of benzylsuccinate synthase and is SAM-dependent [24] and references therein; [26]. NmsA was previously shown to be induced during growth of NaphS2, NaphS3, and NaphS6 on 2-methylnaphthalene [20], and NmsABC from N47, as well as BnsA-H, were shown to be induced in that species during growth on 2-methylnaphthalene [21]. However, NaphS2, NaphS3, and NaphS6 show a considerable lag in growth on 2-methylnaphthalene, which was viewed as evidence that these strains may not use 2-methylnaphthalene as an intermediate during naphthalene degradation [20]. Our microarray and proteomics results are consistent with this observation, since NmsABC and most Bns subunits were not shown to be significantly induced during growth on naphthalene in either microarray or proteomics analyses. The presence of *nms* and *bns* genes in NaphS2 is consistent with its ability to degrade 2-methylnaphthalene, however, the lack of marked induction of these genes or their protein products in our experiments did not indicate a clear role for NmsABC or Bns subunits in degradation of naphthalene.

Previous studies demonstrated a lag in growth when NaphS2 is transferred from naphthalene to 2-methylnaphthalene and further demonstrated that NmsA is induced preferentially on 2-methylnaphthalene [20]. This evidence suggested that 2-methylnaphthalene may not be an intermediate during naphthalene degradation and that NaphS2 may not use methylation as a mechanism for activating naphthalene for degradation, consistent with our results. Thus, we examined the possibility that naphthalene degradation proceeds via carboxylation in NaphS2. A gene cluster upregulated in NaphS2 during growth on naphthalene contained several ORFs with similarity to phenolic acid decarboxylases (NPH_5855, NPH_5857, NPH_5861, NPH_5863). We postulate that these gene products could function to carboxylate naphthalene, though the requirement of these enzymes for a phosphorylated intermediate would call into question whether these carboxylases could carboxylate naphthalene.

### Benzoate Degradation

Approximately 165 genes were upregulated during growth on benzoate versus growth on pyruvate in NaphS2 cultured under anoxic, sulfate-reducing conditions (Table S3). The Ncr gene cluster is the only one identified in the draft NaphS2 genome encoding proteins with similarity to benzoyl-CoA reductase, though there may be other similar genes encoded in gaps within the draft sequence. This gene cluster was not differentially expressed during growth on benzoate versus pyruvate, and upregulation of this gene cluster on naphthalene suggests that it functions as a naphthoyl-CoA reductase. The fact that it is not induced on benzoate suggests that NaphS2 does not use a classical benzoyl-CoA reductase for ring dearomatization of benzoyl-CoA during benzoate degradation, or that if the strain does, the encoded activities are found within a gap in the draft genome sequence. We examined the possibility that other gene clusters are responsible for benzoate degradation in NaphS2.

The dissimilatory Fe(III) reducer *Geobacter metallireducens* is able to couple degradation of benzoate to Fe(III) reduction [22]. Previous studies indicated that *G. metallireducens* does not possess a classical benzoyl-CoA reductase, but instead may utilize a non-classical dearomatizing enzyme during benzoate degradation, designated BamB-BamI, where BamB encodes a novel reductase which may be involved in dearomatization whereas other Bam subunits may play a role in electron transfer during dearomatization [22], [23]). An examination of the NaphS2 genome revealed the presence of two gene clusters with similarity to the *bamB*-*bamI* gene clusters of *G. metallireducens*. The first cluster, NPH_7171-NPH_7176, encodes homologs of BamB-BamF, while a second cluster contains homologs of BamG-BamI (NPH_6169-NPH_6171). Several Bam components were differentially expressed during growth on benzoate at a fold change of greater than or near 2.0 in microarray, and also shown to be induced via proteomics analysis in the insoluble fraction during growth on benzoate versus pyruvate or benzoate versus naphthalene (Figure 1, Table S3-5), suggesting that NaphS2 may couple benzoyl ring dearomatization to electron transfer, as postulated for *G. metallireducens*. Genes within the *bam* clusters were also differentially expressed during benzoate degradation compared to naphthalene degradation, suggesting a role for them exclusively in benzoate degradation (Table S1, 5).

Genes encoding enzymes involved in degradation steps following benzoyl-CoA reduction were also found in the NaphS2 genome and were differentially expressed during growth on benzoate and pyruvate. *bzdY*, encoding a hydrolase involved in a ring cleavage step following benzoyl-CoA reduction, was also upregulated during growth on benzoate versus pyruvate (Table S3, 4), and slightly upregulated (1.7 fold) during growth on benzoate versus naphthalene (Table S3) as was *bzdX*, which encodes a dehydrogenase, and *bzdW*, encoding cyclohexa-1,5-dienecarbonyl-CoA hydratase (Table S3). An ORF with the highest similarity to benzoyl-CoA ligase in NaphS2 (NPH_7071) was not differentially expressed during growth on benzoate, however another long chain fatty acid CoA ligase, NPH_1950, is differentially expressed during growth on benzoate compared versus either pyruvate or naphthalene, suggesting that this NPH_1950 may encode the endogenous benzoyl-CoA ligase in NaphS2. Alternatively, the draft genome sequence may contain a gap in sequence encoding the true benzoyl-CoA ligase. It is also interesting to note that a gene cluster encoding an adenylylsulfate reductase and HdrABC complex was differentially expressed during growth on benzoate versus pyruvate, and it was not differentially expressed during growth on naphthalene versus benzoate, suggesting that the rate of sulfate reduction may increase during growth on aromatic compounds compared to during growth on C3 compounds, and raising the possibility that components of this gene cluster may be useful for indirectly tracking aromatic compound degradation by assaying the rate of activity of these enzymes.

Proteomic analysis using FTICR-MS was used to confirm microarray and RT-PCR results for growth of NaphS2 on benzoate. During growth on benzoate versus pyruvate, the NcrA (NPH_5890) and NcrB (NPH_5891) subunits of the putative benzoyl-CoA reductase were upregulated in the insoluble fraction. Also upregulated during growth on benzoate in the insoluble fraction were putative BamB ferredoxin:oxidoreductase (NPH_7171), as well as BamC (NPH_7173) and BamE (NPH_7175). In addition BzdX (NPH_5404) and BzdY (NPH_5405) were upregulated during growth on benzoate versus growth on pyruvate in the soluble fraction. BzdW and BzdZ were both upregulated on benzoate in the insoluble pellet. In the soluble fraction, BzdX (NPH_5404) and BzdW (NPH_5406) were upregulated during growth on benzoate as was a predicted 4-hydroxybenzoate-CoA reductase subunit (NPH_3309). Moreover, proteins encoded by genes downstream of benzoyl-CoA reductase genes were also upregulated (NPH_5893, NPH_5895, and NPH_5899).

### Toluene Degradation

**Figure 4:**
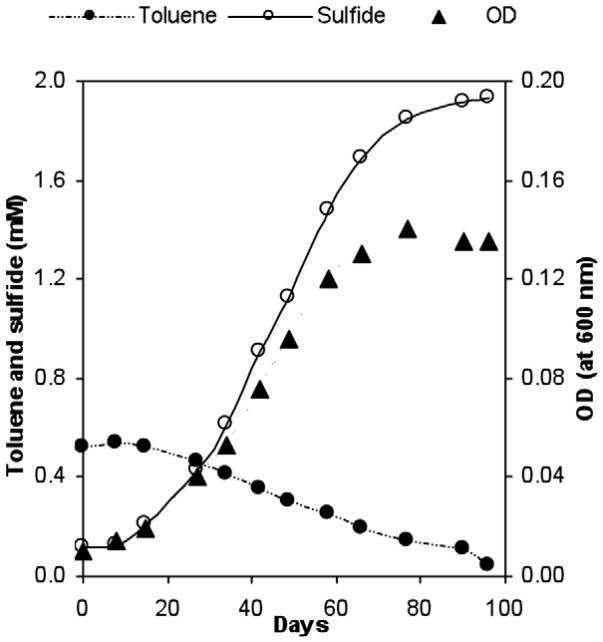
NaphS2 degrades toluene. Increase in cell density and sulfide concentration during anaerobic growth of strain NaphS2 with toluene. The growth experiment was performed in a 150 ml serum bottle containing 100 ml of medium and toluene dissolved in heptamethylnonane.

Because homologs of the toluene degradation genes *bss* and *bbs* (named *nmsABC* and *bnsA-H* for their N47 homologs, Table S2) were identified in the draft NaphS2 genome, the question of whether NaphS2 can metabolize toluene was investigated by inoculating NaphS2 into medium that contained toluene (500 µM) as the sole electron donor (Figure 4). NaphS2 grew and could be continually transferred and propagated in this medium, and the consumption of toluene and the production of sulfide were consistent with toluene oxidation coupled to sulfate reduction when it is considered that small amounts of toluene would be required for cell carbon, while cultures amended with higher toluene concentrations did not grow. Future experiments will be necessary to determine whether these genes are induced during growth on toluene, and therefore likely involved in toluene degradation, or whether another cluster, present within a gap within the draft genome sequence, is responsible.

### Other Monoaromatics

The genome sequence of NaphS2 also suggests a capacity to degrade other monoaromatic compounds, including phenol, p-cresol, benzyl alcohol, and 4-hydroxybenzoate. NPH_5855, NPH_5859, NPH_5861, and NPH_5863 are a cluster of four genes, encoding phenylphosphate carboxylase subunits, with similarity to *ppcA* and *ppcB* from *Thauera aromatica*. In *T. aromatica*, phenylphosphate carboxylase is encoded by *ppcBCAX*, whereas the NaphS2 cluster consists of genes more similar to *ppcA* and *ppcB* than to *ppcC* and *ppcX*. Homologs to *Pseudomonas putida* p-cresol methylhydroxylase, *pchCF*, are also present in the genome (NPH_3625, NPH_5226). Benzyl alcohol dehydrogenase is coded by the *Aromatoleum aromaticum* EbN1 gene *adh* (ebA3166), of which NPH_4462 is a homolog, whereas NPH_5226 is a homolog of the EbN1 benzaldehyde dehydrogenase ebA5642. NPH_7071 encodes a homolog to *T. aromatica* 4-hydroxybenzoate:CoA ligase, whereas NPH_2861-63 encode subunits of 4-hydroxybenzoyl-CoA oxidoreductase.

## Discussion

### Naphthalene Degradation

Consistent with protein expression results from a previous study [20], the gene encoding the proposed catalytic subunit of the 2-methylnaphthalene-activating enzyme (2-naphthylmethyl)succinate synthase, NmsA, was not upregulated during growth on naphthalene vs. benzoate, though it did show an induction of below 2-fold during growth on naphthalene vs. pyruvate. Although BnsD, a putative naphthyl-2-hydroxymethyl-succinyl-CoA dehydrogenase subunit, and BnsG, a putative naphthyl-2-methyl-succinyl-CoA dehydrogenase, were shown to be expressed during growth on naphthalene in proteomics analysis, NmsABC and most Bns subunits, and their corresponding genes, were not shown to be significantly induced during growth on naphthalene in either microarray or proteomics analyses. This suggests that NmsABC and most Bns subunits do not play a role in naphthalene degradation in NaphS2, while we would expect them to play a role in 2-methylnaphthalene degradation. The fact that BnsD and BnsG are upregulated during growth on naphthalene leaves open the possibility that methylation is used to activate naphthalene in NaphS2, however as mentioned previously, the conflicting observation that NmsABC was not upregulated during growth on naphthalene in our experiment and previous experiments raises doubts regarding that hypothesis. Our studies indicate that NaphS2 may activate naphthalene in manner such as carboxylation or by some other mechanism, but uses NmsABC and Bns subunits to specifically degrade 2-methylnaphthalene. Due to the draft nature of the NaphS2 genome, we cannot rule out that other gene clusters encoding NmsABC and other *bns* genes may exist within a gap in the genome. However, available genome and experimental evidence to date suggests that methylation is not the initial step in naphthalene degradation. Future experiments should compare expression of *nmsABC* and *bns* genes during growth on 2-methylnaphthalene and naphthalene.

A lack of clear induction of genes in NaphS2 that have previously been shown to be involved in 2-methylnaphthalene degradation in N47 led us to examine the possibility that naphthalene degradation proceeds via carboxylation. Three genes encoding probable aromatic carboxylases were upregulated during growth on naphthalene vs. benzoate (*NPH_5859, NPH_5863* and *NPH_5865*). One or more of these genes may carboxylate NaphS2, or naphthalene activation may occur through a different set of enzymes, or by an as of now unidentified mechanism other than methylation or carboxylation.

According to the pathway suggested by Annweiler [16], the next two steps are ring reductions (Figure 2). Two ring reductases are up-regulated during growth on naphthalene vs. benzoate (Figure 1).

One hypothesis is that the products of the novel reductase genes (NPH_5472-76) reduce the first ring of the double naphthalene ring (since it is most unique structurally, relative to benzoate) and then the products of *bcr*-like genes (NcrABCD) reduce the second ring in manner analogous to benzoate degradation. The subsequent ring opening steps might then use the same enzymes as the analogous reactions in the benzoate degradation pathway.

### Aromatic Ring Reduction

Draft genome evidence suggests that strain NaphS2 has three sets of genes potentially involved in the reduction of aromatic rings. Two of these sets are similar to enzymes involved in the reduction of the monoaromatic compound benzoate. There is an operon, *bamBCDEFGHI*, that is homologous to the putative benzoate reductase from the obligate anaerobes *G. metallireducens* and *D. multivorans*. In NaphS2, both gene and protein expression levels of BamB-I are increased during benzoate metabolism and decreased during naphthalene metabolism, although some expression fold changes were below 2-fold. This suggests that this enzyme may be used specifically for benzoate metabolism, while another enzyme (NcrABCD) reduces the monoaromatic ring during naphthalene metabolism, and that downstream components of this gene cluster encode enzymes capable of later degradation steps following the ring reduction events of anaerobic naphthalene.

The Bzd enzyme has been shown to require two low-potential ferredoxins and two ATPs to reduce benzoyl-CoA in the nitrate reducers[27]. The use of a different enzyme for this reaction in species like *G. metallireducens* which does not use nitrate has been attributed to the lower energy yield available from their metabolism[23]. The change in expression between the two putative benzoate reductases in NaphS2 may be due to a difference in energy yield between naphthalene and benzoate degradation. Sequence analysis suggests that the use of ferredoxin may be involved in adapting to these yield changes.

An additional reductase would be required to reduce the first aromatic ring of naphthalene, but would be unnecessary during benzoate metabolism. The third putative reductase, NPH_5472-NPH_5476, is one of the most highly upregulated clusters during naphthalene degradation, but shows little or no upregulation during benzoate metabolism. Thus, this enzyme may be involved in the reduction of the first aromatic ring of naphthalene.

### Toluene Degradation

**Figure 5:**
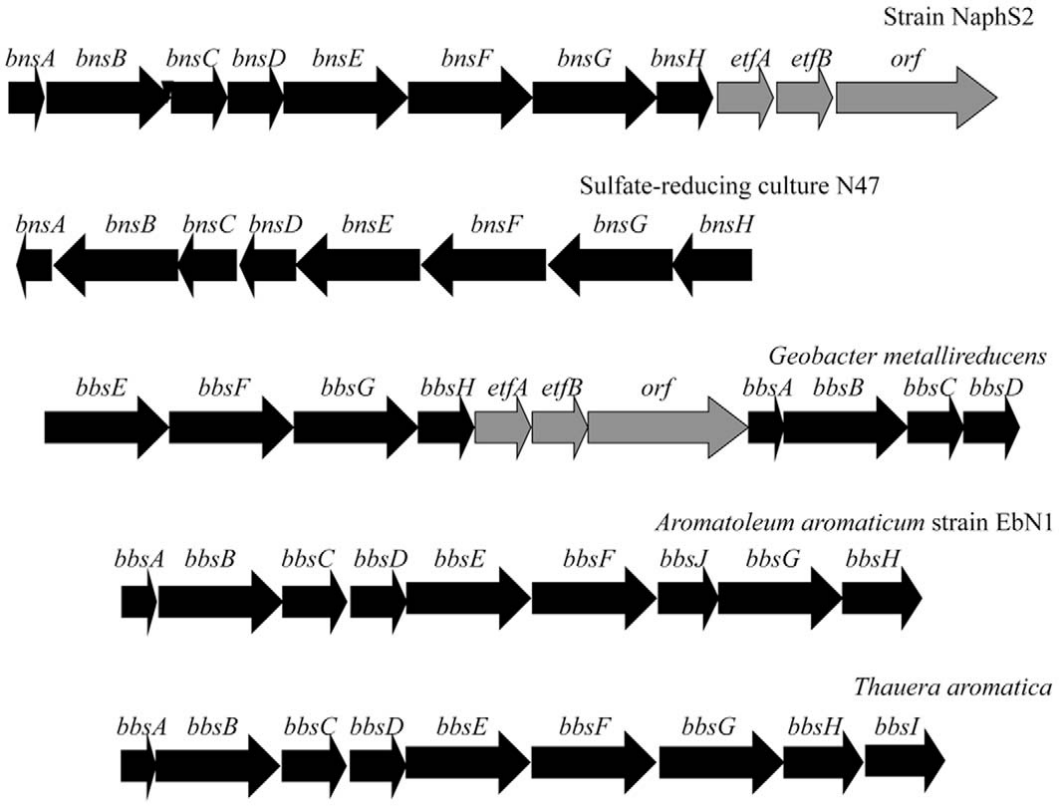
The bnsA-H gene cluster in NaphS2 and N47 and the homologous BbsA-H clusters from other species. Arrangement of bnsA-H genes in strain NaphS2 compared to sulfate-reducing culture N47 and other species. The arrangement of the cluster is similar to EbN1, however genes downstream of *bbsH* in G. metallireducens are also present downstream of *bnsH* in NaphS2.

Prior to completion of the draft NaphS2 genome sequence, the presence of gene clusters similar to *bss* and *bbs* gene clusters (Figure 5) involved in toluene degradation, and isolated through shotgun sequencing, led us to speculate that NaphS2 might be capable of toluene degradation. This has now been shown experimentally. The presence of a gene cluster, encoding Bss and Bbs homologs, *nmsABC* and *bnsA-H*, suggests that toluene degradation might proceed through the same mechanism as 2-methylnaphthalene degradation in NaphS2, namely through Nms and Bns, however we cannot rule out the presence of an additional set of *bss* and *bbs* gene clusters within a gap in the NaphS2 genome. While further work is necessary to determine whether the *nmsABC* and *bnsA-H* gene clusters are upregulated during growth on toluene, the ability of NaphS2 to grow on toluene demonstrates that genome sequencing may provide additional insights into substrate utilization.

### Limits of Identifying Degradative Pathway Genes using a Draft Genome Approach

This study presents the draft genome of the naphthalene degrading, marine sulfate reducer NaphS2. The combined strategy of draft genome sequencing, microarray analysis, and proteomic analysis provide a powerful approach toward elucidating naphthalene degradation pathways in NaphS2; however, the approach is not without limitations. This study has attempted to identify key genes involved in the anaerobic degradation of naphthalene, however given that gaps are more than likely present within the draft genome of NaphS2, we cannot rule out the existence of certain enzyme activities in NaphS2 on the basis of this approach, or that additional enzyme activities do not also contribute to naphthalene degradation in this organism. The availability of a finished genome, enzymatic assays on select enzymes identified as likely candidates in naphthalene degradation, and complementary whole genome expression and proteomics approaches will shed additional light on aspects of naphthalene degradation which remain unclear, particularly regarding the initial activation of naphthalene for degradation. While we have identified candidates for certain enzymatic activities based on their induction in microarray and proteomics experiments and their predicted enzymatic activities, particularly in regard to carboxylation of NaphS2 and alternative mechanisms of ring-opening during naphthalene degradation, many other hypothetical genes or genes with predicted roles in aromatic hydrocarbon degradation may also play key roles in naphthalene degradation in NaphS2.

## Materials and Methods

### Culturing NaphS2

Benzoate-degrading, sulfate-reducing strain NaphS2 [15] was obtained from Deutsche Sammlung von Mikroorganismen (Braunschweig, Germany; DSM 14454) and cultured anaerobically in defined bicarbonate-buffered, strictly anoxic artificial seawater medium containing sulfate as described previously [28]. Nonchelated trace element solution[28] and sodium selenite were added to promote bacterial growth. Sodium dithionite was added as additional reductant before inoculation. The culture medium and cultures were kept serum vials under a headspace containing an N_2_-CO_2_ mixture (80∶20, vol/vol), sealed with a butyl rubber stopper.

Filter-sterilized (solvent-resistant cellulose filters; pore size, 0.2 µm) substrates (purity, >99%) and other hydrocarbons were dissolved in sterile, anoxic 2,2,4,4,6,8,8-heptamethylnonane (HMN) as the carrier phase to avoid toxic effects of the pure substances [29], [30]. The sealed culture vials were incubated nearly horizontally to facilitate diffusion of the added hydrocarbons into the aqueous medium and the level of the medium was adjusted to avoid contact of the medium with the stoppers. All incubations were under anoxic conditions at 30°C in the dark.

### Genome Sequencing and Annotation

The genome of NaphS2 was sequenced to a draft stage by the whole random shotgun method [31], [32]. Briefly, one small insert plasmid library (2–3 kb) and one medium insert plasmid library (8–10 kb) was constructed by random nebulization and cloning of genomic DNA. The small insert library was constructed using a pUC18 vector (http://www.jgi.doe.gov/sequencing/protocols/prots_production.html). The medium sized insert library was constructed using a pHOSII cloning vector. In the initial random sequencing phase, approximately 6-fold sequence coverage was achieved from the two libraries (each library was sequenced to 3-fold coverage, respectively). The average read sizes for the small and large insert libraries were 831 nt and 817 nt respectively. The sequences were assembled using the Celera Assembler [33]. A pseudomolecule was constructed in which gaps between contigs were filled with a 36 bp linker sequence designed to produce a stop codon in all six reading frames. An initial set of ORFs that likely encode proteins was identified using GLIMMER [34]. The ORF prediction and gene family identifications were completed using the methodology described previously [31], [32]. Two sets of hidden Markov models (HMMs) were used to determine ORF membership in families and superfamilies. These included HMMs from Pfam v22.0 [35] and from the TIGR ortholog resource [36]. TMHMM [37] was used to identify membrane-spanning domains (MSD) in proteins. Additional attributes of biological function were obtained by classifying genes within Clusters of Orthologous Groups (COG) and by assignment of GeneOntology terms and their corresponding numbers. Putative functional role categories were assigned as previously described [31], [32].

The nucleotide sequence and the corresponding auto-annotations for the draft genome of NaphS2 have been deposited at DDBJ/EMBL/GenBank under the accession ADZZ00000000. The version described in this paper is the first version, ADZZ01000000.

### Microarray Analysis

For microarray analyses triplicate cultures of NaphS2 were grown with either benzoate (5 mM), naphthalene (3 mM), or pyruvate (10 mM) as the electron donor and sulfate (20 mM) as the electron accetpor. Cells were harvested with centrifugation and the cell pellets frozen. Total RNA was extracted from frozen cell pellets with the Qiagen RNAeasy midi kit according to manufacturer's instructions (Qiagen, Inc.). RNA was DNase-treated with the Ambion DNA-free kit (Ambion, Austin, TX) and tested for genomic DNA contamination by polymerase chain reaction for 40 cycles (96°C 3 min; 94°C 15 sec, 55°C 30 sec, 72°C 90 sec, 40 cycles; 72°C 10 min) using primers specific to NaphS2. 200 ng of total RNA was amplified with the MessageAmpII-Bacteria RNA Amplification kit (Ambion, Austin, TX), ethanol precipitated, and resuspended to ∼1 µg/µl in nuclease-free H_2_O (Ambion, Austin, TX). 10 µg of RNA was chemically labeled with Cy3 (control) or Cy5 (experimental) dye with the MicroMax ASAP RNA Labeling Kit (Perkin Elmer, Wellesley, MA). 2 µl of Cy3 or Cy5 reagent and 6 µl of ASAP labeling buffer were added to 10 µg of RNA in a 20 µl total volume and heated at 85°C for 15 min. The subsequent labeled RNA was resuspended in 200 µl of H_2_O and washed 3X with 200 µl H_2_O using MicroCon spin columns (Millipore, Bedford, MA). RNA from pyruvate grown cultures (control) and RNA from either benzoate or naphthalene grown cultures (experimental) was then combined, ethanol precipitated, and resuspended to 20 µl in nuclease-free H_2_O.

Two replicate hybridizations (technical replicates) were performed for each biological replicate (three biological replicates). Combimatrix (Seattle, WA) 12K CustomArray microarray slides [38] were designed using Combimatrix oligo design algorithms based on the entire set of predicted ORFs from the NaphS2 genome. Duplicate oligos were filtered out of the array design, and the genome was split into two arrays due to its size. Slides were incubated in 100 µl H_2_O (in hybridization chambers) in a rotisserie hybridization oven for 10 min at 65°C to remove oxygen from the slides. Slides were then incubated at 45°C for 30 minutes in prehybridization solution (6X SSPE [Ambion], 0.05% Tween-20, 20 mM EDTA [Ambion], 5X Denhardt's solution [Sigma Chemical Co.], 100 ng/µl salmon sperm DNA [Sigma], 0.05% SDS [Ambion]). After labeling and fragmentation, RNA was added to hybridization buffer (100 µl total volume, 6X SSPE, 0.05% Tween-20 [Sigma], 20 mM EDTA [Ambion], 25% formamide [Ambion], 100 ng/µl salmon sperm DNA [Sigma], 0.05% SDS [Ambion]) heated at 95°C for 3 minutes, chilled briefly on ice, added to microarray slides, and incubated at 45°C overnight. Slides were washed for 6 successive washes of 5 minutes in the following solutions: Wash I (6X SSPE, 0.05% Tween-20), Wash II (3X SSPE, 0.05% Tween-20) Wash III (0.5X SSPE, 0.05% Tween-20), Wash IV (2X PBS [Sigma], 0.1% Tween-20), and Wash V (2X PBS) twice. The first wash was at 45°C, and all subsequent washes were at room temperature. Slides were then immersed in visualization solution, covered with a LifterSlip, scanned using a GenePix 4000B scanner (Axon Instruments, Inc., Union City, CA), and analyzed using GenePix and Acuity 4.0 software (Axon Instruments, Inc.).

Arrays were scanned with Genepix software version 4.0. The total intensity from each spot for each channel was calculated. Data obtained were then exported into the R package (www.r-project.org) for further analysis as described previously [38].

A background calculation was derived in order to allow normalization of data across arrays. This calculation was based on the signals from a set of negative control spots on the array containing oligos from humans or plants that should not hybridize to bacterial RNA. For each channel of each array, the signal intensities from the lowest third of the negative control spots were averaged and the standard deviation was calculated. Global background subtraction was performed by subtracting the mean background intensity plus two standard deviations from the signal of all experimental spots. Low intensity spots having background-corrected intensity less than three times the background standard deviation in both channels were excluded from further analysis.

For the remaining data, the logged ratios [M = log2(WT/EX)] are then calculated and lowest global normalization are performed. During the data preprocessing, M vs A plots, before and after normalization, and side-by-side boxplots for all arrays are used to assess array quality. LIMMA mixed model analysis (R-package LIMMA) was applied as described previously [38]. To limit false positives, a gene was called differentially expressed if at least half of its probes met a Limma P-value cut-off of 0.001 [38].

### Proteomic Analysis

FTICR-MS analysis was conducted on soluble and insoluble fractions of three biological replicates analyzed in duplicate for each condition (naphthalene, benzoate, and pyruvate), and all protocols relating to protein isolation, FTICR-MS analysis, and computational methods to generate raw protein values, zeroed abundance values, and Z-score for each protein may be found in Methods S1. Proteins displaying significant differences in expression between either benzoate versus pyruvate, naphthalene versus pyruvate, or naphthalene versus benzoate were identified by t-test (p-value cut-off 0.01) using MeV software, in which log_2_ transformed, zeroed protein values were analyzed by t-test between two conditions, and the average Z-scores for these statistically significant proteins were calculated for each condition. Results of t-test analysis, including mean log transformed zeroed protein abundance value, and average Z-score for proteins (p-value cut-off 0.01), were determined for soluble and insoluble fractions.

### Real Time PCR Primer Design and Quantification of mRNA Gene Transcripts

Gene-specific primers were designed for quantitative PCR analysis from the NaphS2 genome using the Primer Express software (ABI). Optimal primers pairs were checked for dimers and hairpins using the NetPrimer software (PREMIER Biosoft, Palo Alto, CA), optimized using PCR gradient analysis, cloned using the TOPO p2.1 kit (Invitrogen Corp., Carlsbad, CA), and sequenced to verify gene specificity. Sample cDNA was generated from mRNA transcripts using the DuraScript Enhanced Avian RT single-strand synthesis kit (Sigma Aldrich) according to the manufacturer's instructions. Positive RT reactions were verified using PCR and visualized on a 2% agarose gel stained with ethidium bromide.

The number of mRNA transcripts was quantified using the Applied Biosystems 7500 Real-Time PCR system (PE Biosystems, Foster City, CA). Reactions (25 µl total volume) consisted of 12.5 µl 2× POWR SYBR green master mix (Applied Biosystems), 5 µl of 1∶10 diluted template cDNA, and 200 pmol of the appropriate primer pair. The thermal cycling parameters consisted of an activation step of 50°C for 2 min, a denaturation step of 95°C for 10 min, followed by 45 cycles at 95°C for 15 s and 60–66°C for 1 min. A standard curve and two types of negative controls were run concurrent with cDNA samples. Standard curves covered a range of approximately 8 orders of magnitude and were constructed as previously described [39].The first negative controls contained RNA template from each sample that had not been subjected to RT, and the second controls contained reaction solution without template. Real-time PCR product size and amplification was verified on a 2% agarose gel stained with ethidium bromide.

### Toluene Degradation by NaphS2

NaphS2 cells were grown as above, but were adapted to growth with sulfate (20 mM) as electron acceptor and toluene (0.5 mM) as electron donor over at least five passages prior to conducting the growth studies reported here. Filter-sterilized (solvent-resistant cellulose filters; pore size, 0.2 µm) toluene (purity, >99%) and other hydrocarbons were dissolved in sterile, anoxic 2,2,4,4,6,8,8-heptamethylnonane (HMN) as the carrier phase to avoid toxic effects of the pure substances [29], [30].

The time course of sulfate reduction to sulfide with toluene as the electron donor was measured in the flat bottles described above. Aliquots for determination of the optical density at 600 nm and sulfide and sulfate content were withdrawn from the closed bottles via the stoppers by using sterile syringes.

Toluene in heptamethylnonane and in the aqueous phase was measured by gas chromatography on a Hewlett-Packard series 1100 **(**Agilent Technologies, Inc., Santa Clara, CA). Sulfide was determined colorimetrically as described previously [40], [41]. Sulfate was measured with a Dionex ICS 2000 ion chromatograph (Dionex Corp., Sunnyvale, CA).

## Supporting Information

Table S1 Genes Upregulated During Growth on Naphthalene versus Growth on Benzoate.
(0.08 MB XLS)

Table S2 The best two BLAST hits of some NaphS2 genes. Blastp results, excluding hits with unknown strain information.
(0.04 MB XLS)

Table S3 Genes Upregulated During Growth on Benzoate versus Growth on Pyruvate.
(0.08 MB XLS)

Table S4 Proteomics Results, Soluble Fraction.
(0.03 MB XLS)

Table S5 Proteomics Results, Insoluble Fraction.
(0.03 MB XLS)

Table S6 Genes Upregulated on Naphthalene vs Pyruvate.
(0.11 MB XLS)

Methods S1 (0.04 MB PDF)
