# Supplementary material for: Genome Sequence of the Deltaproteobacterial Strain NaphS2 and Analysis of Differential Gene Expression during Anaerobic Growth on Naphthalene
Source: PLoS One. 2010 Nov 19;5(11):e14072. doi: 10.1371/journal.pone.0014072 (PMC2988823; doi:10.1371/journal.pone.0014072)
Supplement: Methods S1 — (0.04 MB PDF) [file pone.0014072.s007.pdf]

## Supplemental Methods

## FTICR-MS analysis

**Culture Conditions and Cell Lysis.** The following chemicals used, unless otherwise noted, were obtained from the Sigma-Aldrich Company (St. Louis, MO) and were of analytical grade. Cells were resuspended in a volume of nanopure water which was 2 x the volume of the cell pellet and lysed with a bead beater. The cells were vortexed into a suspension and were beadbeated with 0.1 mm zirconia/silica beads in a mini-beadbeater (Biospec, Bartlesville OK) for 90 s at 4500 rpm. Lysates were collected and placed immediately on ice to inhibit proteolysis.

**Cleared Lysate and Pellet Fractionation.** An ultracentrifugation was employed to separate out the dirt like pellet from the soluble proteins released after lysis. To each cell lysate obtained from the procedure above, water was added in a volume that was 10 x that of the starting lysate. The dilute lysates were then ultracentrifuged at 100 000 rpm (355 040 rcf) with a Beckman (Fullerton, CA) Optima TL ultracentrifuge for 20 min. The pellet was the insoluble fraction and the supernatant was the soluble fraction.

**Protein Mass Determination and Addition of an Internal Protein Standard.** The insoluble pellets obtained from above were made suspended into solution by adding two pellet volumes of nanopure water to each pellet followed by sonication. The protein concentration of each whole cell lysate, and subcellular fraction (supernatant and pellet/membrane) were measured using a Coomassie Plus protein assay (Pierce, Rockford, IL) using a bovine serum albumin standard. A measured volume of each

sample was transferred to a new 1.5 mL microfuge tube and thus the total protein mass was known for each tube. 1 mg of apomyoglobin (equine), 0.363 mg of cytochrome c (bovine) and 0.210 mg of G-2-PHD (rabbit) was added to 1 mL total volume of nanopure water. This was the internal protein reference standard used in the samples to ensure that replicate analysis and instrument performance in time was consistent. To each whole protein samples, supernatant (cleared lysate protein) fractions, and pellet (membrane protein) fractions, 10 µL (15.73 µg) of the internal protein reference was added to every 984 µg of sample.

**Trypsin Digestion.** The cleared lysates were digested with trypsin as described elsewhere (*Masselon, C.; Pasa-Tolic, L.; Tolic, N.; Anderson, G. A.; Bogdanov, B.; Vilkov, A. N.; Shen, Y.; Zhao, R.; Qian, W. J.; Lipton, M. S.; Camp, D. G., 2nd; Smith, R. D. Targeted Comparative Proteomics by Liquid Chromatography-Tandem Fourier Ion Cyclotron Resonance Mass Spectrometry. Anal. Chem.* ***2005****, 77 (2), 400-6*). To the pellets from the ultracentrifugation, the same digestion was carried out with the exception that 4% (w/v) CHAPS was also included in the denaturation solution.

**Peptide Concentration and Cleanup.** The cleared lysate digests were desalted using Supelco (St. Louis, MO) Supelclean C-18 tubes as described elsewhere.(*Masselon, C.; Pasa-Tolic, L.; Tolic, N.; Anderson, G. A.; Bogdanov, B.; Vilkov, A. N.; Shen, Y.; Zhao, R.; Qian, W. J.; Lipton, M. S.; Camp, D. G., 2nd; Smith, R. D. Targeted Comparative Proteomics by Liquid Chromatography-Tandem Fourier Ion Cyclotron Resonance Mass Spectrometry. Anal. Chem.* ***2005****, 77 (2), 400-6*). CHAPS will bind to C-18 and elute with peptides, (*Hixson, K. K.; Rodriguez, N.; Camp, D. G., 2nd; Strittmatter, E. F.; Lipton, M. S.; Smith, R. D. Evaluation of Enzymatic Digestion and Liquid Chromatography-mass Spectrometry Peptide Mapping of the Integral Membrane Protein Bacteriorhodopsin. Electrophoresis* ***2002****, 23 (18), 3224-3232).* thus Supelco Supelclean SCX tubes were used to desalt the pellet/membrane fraction digest. The

digestion mixture pH was adjusted to 3.5 by the addition of dilute acetic acid and an equal volume of 10 mM ammonium acetate, pH 3.5 with 25% acetonitrile. The resin was conditioned with one column volume of acetonitrile followed by one column volume of 25% acetonitrile in 10 mM ammonium acetate, pH 3.5. After the peptide mixtures were loaded onto the resin, the peptides were washed with six column volumes of the same 25% acetonitrile in 10 mM ammonium acetate, pH 3.5. Peptide elution was accomplished with one column volume of 35% acetonitrile in 500 mM ammonium acetate, pH 8.5, followed by 100% acetonitrile. All eluted peptides were concentrated via speedvac (ThermoSavant, San Jose CA) until protein concentrations were 1.0 mg/mL. Peptide concentrations were determined by BCA assay (Pierce, Rockford IL) with a bovine serum albumin standard.

**SCX Fractionation of Peptides for Potential Mass and Time (PMT) Tag Aquisition.** 300 μg each of a pooled sample from the pellets above and a pooled sample from the cleared lysates from above were fractionated with a strong cation exchange (SCX) fractionation as described elsewhere. (*Qian, W. J.; Liu, T.; Monroe, M. E.; Strittmatter, E. F.; Jacobs, J. M.; Kangas, L. J.; Petritis, K.; Camp, D. G., 2nd; Smith, R. D. Probability-Based Evaluation of Peptide and Protein Identifications From Tandem Mass Spectrometry and SEQUEST Analysis: The Human Proteome. J. Proteome Res.* ***2005****, 4 (1), 53-62*). These fractions were analyzed by an ion trap in MS/MS mode as described below.

**Capillary LC Separations.** All peptide mixtures from the whole cell lysate, supernatant, and pellet fractions as well as the whole cell lysate SCX fractions were then separated by an automated in-house designed HPLC system as described elsewhere *(Masselon, C.; Pasa-Tolic, L.; Tolic, N.; Anderson, G. A.; Bogdanov, B.; Vilkov, A. N.; Shen, Y.; Zhao, R.; Qian, W. J.; Lipton, M. S.;*

*Camp, D. G., 2nd; Smith, R. D. Targeted Comparative Proteomics by Liquid Chromatography-Tandem Fourier Ion Cyclotron Resonance Mass Spectrometry. Anal. Chem.* ***2005****, 77 (2), 400-6; Shen, Y.; Tolic, N.; Zhao, R.; Pasa-Tolic, L.; Li, L.; Berger, S. J.; Harkewicz, R.; Anderson, G. A.; Belov, M. E.; Smith, R. D. High-throughput Proteomics Using High-efficiency Multiple-capillary Liquid Chromatography with On-line High-Performance ESI FTICR Mass Spectrometry. Anal. Chem.* ***2001****, 73 (13), 3011-21)* and eluted directly into either an ion trap (for MS/MS as described below) or an FTICR MS (as described below).

**Potential Mass and Time Tag (PMT) Acquisition.** The eluate from the HPLC was directly electrosprayed into an ion trap MS (LCQ, ThermoFinnigan, San Jose, CA) using electrospray ionization (ESI). For the MS/MS detection, a total of 10 µ (1 µg/µL) of total peptide were loaded onto the reversed phase column for each analysis. The mass spectrometer operated in a data-dependent MS/MS mode for the whole cell lysate, supernatant fraction, and pellet fraction trypsin digests from all analyzed samples. The peptide fractions produced from the SCX peptide separations were analyzed with one full *m*/*z* range (400-2000) each. The details for PMT generation are described elsewhere. (*Smith, R. D.; Anderson, G. A.; Lipton, M. S.; Pasa-Tolic, L.; Shen, Y.; Conrads, T. P.; Veenstra, T. D.; Udseth, H. R. An Accurate Mass Tag Strategy for Quantitative and High-throughput Proteome Measurements. Proteomics* ***2002****, 2 (5), 513-23.*) The MS/MS spectra were analyzed using the peptide identification software SEQUEST (*Eng, J. K.; McCormack, A. L.; Yates, J. R. An Approach to Correlate Tandem Mass Spectral Data of Peptides with Amino Acid Sequences in a Protein Database. J. Am. Soc. Mass Spectrom.* ***1994****, 5 (11), 976-989*) in conjunction with the annotated protein translations from the genome sequence of *Delta proteobacterium NaphS2*. Qualitative/cursory identifications in the putative mass and elution time (PMT tag) peptide library were based on a minimum cross correlation (Xcorr) score of 1.5 for all peptides identified at least twice in all MS/MS experiments. For peptides only identified once, Xcorr values had to be a minimum of 1.9, 2.2, and 3.5

for charge states of 1+, 2+, and 3+ respectively. When the data were analyzed against a reverse database, the false positive rates were 5% or less.(*Qian, W. J.; Liu, T.; Monroe, M. E.; Strittmatter, E. F.; Jacobs, J. M.; Kangas, L. J.; Petritis, K.; Camp, D. G., 2nd; Smith, R. D. Probability-Based Evaluation of Peptide and Protein Identifications From Tandem Mass Spectrometry and SEQUEST Analysis: The Human Proteome. J. Proteome Res.* ***2005****, 4 (1), 53-62* ) A mass tag database creation tool called MTDB Creator is available at http://ncrr.pnl.gov/software/MTDBCreator.stm.

**Accurate Mass and Time (AMT) Tag Identification and Alignment.** Using 3 µg (0.5 µg/µL) of total peptide from each of the same samples analyzed above by MS/MS, intact peptide mass data were obtained using the same HPLC system but using an FTICR mass spectrometer for detection. A mass calibration mixture was infused at the end of each analysis, and the masses of the compounds in the mixture were used to calibrate all of the spectra within the analysis. The PMT tags identified from the tandem mass spectrometry analyses were next matched against unique peptides detected with the FTICR by the means stated next. Data from each FTMS analysis was deisotoped using the in-house software tool ICR-2LS (http://ncrr.pnl.gov/software/ICR2LS.stm). Chromatographic peak profiles of different unique deisotoped masses were discovered and grouped by using the VIPER (http://ncrr.pnl.gov/software/VIPER.stm) software package which looks for similar masses over consecutive scans and groups them together if they are within a specified mass tolerance of each other. As a result of these two steps the LC-MS experiment data were collapsed into a list of monoisotopic masses and the elution time centers of isotopic signatures of chemical species seen in the LC-MS experiments. Each of these mass and elution time centers is referred to as a *feature* and conceptually corresponds to a peptide whose isotopic profile is observed in the mass spectra over

multiple scans. Also available for each feature is its abundance from the mass spectra it was observed in during the analyses. Details of the algorithms involved in this process are available elsewhere (*Zimmer, J. S. D.; Monroe, M. E.; Qian, W. J.; Smith, R. D., Advances in proteomics data analysis and display using an accurate mass and time tag approach. Mass Spectrometry Reviews 2006, 25, (3), 450-482*).

Features in multiple FTICR MS datasets with similar mass and elution times are presumed to represent the same chemical species in different analyses. By collating features from the same chemical species into a master feature, we can study how its abundance is distributed in replicates from one sample and compare it against the abundance in the other samples in which a comparison is desired. However, before such features can be grouped into a master list on the basis of mass and elution time, we must remove variability in mass measurement accuracy and elution time in the different analyses. To do so, the datasets were all first aligned in mass and elution time to one baseline experiment using the LCMSWARP algorithm (*Zimmer, J. S. D.; Monroe, M. E.; Qian, W. J.; Smith, R. D., Advances in proteomics data analysis and display using an accurate mass and time tag approach. Mass Spectrometry Reviews 2006, 25, (3), 450-482*) which is a component of the in-house developed software MultiAlign (http://ncrr.pnl.gov/software/) which finds a global transformation of elution time and mass measurement accuracy which results in the highest similarity of the aligned subsections of all experiments. After alignment of all datasets to a baseline dataset, the aligned elution times are normalized to values between 0 and 1 and referred to as the normalized elution time (NET).

After alignment of all datasets to one baseline, the master list of features is created using a clustering algorithm which groups together features of similar mass and elution time, with single linkage clustering using a mass tolerance of ±8 ppm and an

elution time tolerance of ±0.05 (normalized elution time). Each group of features is referred to as a cluster. A clusters' monoisotopic mass and NET are calculated as the average of the monoisotopic mass and NETs of the features observed for it in the datasets. Each cluster found is inferred to be a compound (most probably a peptide) which was seen in different experiments as features with similar mass and elution time values. We refer to the clusters interchangeably as features themselves, because conceptually they are the same thing. As mass spectral data are intrinsically noisy, we removed all clusters which were not observed with at least two features in any two samples.

The aligned clusters/features that also match with regard to NET, mass and which pass the cutoff filters of a minimum Sequest Xcorr score of 2, a minimum discriminant score of 0.6 (*Strittmatter, E. F.; Kangas, L. J.; Petritis, K.; Mottaz, H. M.; Anderson, G. A.; Shen, Y.; Jacobs, J. M.; Camp, D. G. I.; Smith, R. D. Application of Peptide Retention Time Information in a Discriminant Function for Peptide Identification by Tandem Mass Spectrometry. J. Proteome Res.* ***2004****, 3 (4), 760-769*), and a Peptide Prophet P value of 0.9 (http://www.proteomesoftware.com/Proteome_software_pro_pep_prophet.html) are identified as accurate mass and time (AMT) tags. These AMT tags list the peptide identified which corresponds to an abundance value which is obtained from the summed peptide ion current intensity from all MS scans in which it elutes. So in the end you have the peptide identity and an abundance value for each peptide identified in each analysis.

**Normalization of Replicate Analyses.** Each sample was analyzed three times. The samples were run in an order that was blocked in a Latin Square Design with randomization to limit instrument or sample order biases. After peak matching and

alignment using MultiAlign (http://ncrr.pnl.gov/software/), the peptide abundance values across replicates were normalized using a linear regression normalization using reference dataset created by calculating the median value for each peptide for all equivalent replicates.

**Protein Roll-up from Peptide Values.** From the normalized peptides abundance values, a protein abundance value was obtained by using a reference peptide from each protein observed. This value then represented the protein abundance value for each protein in each run.

**Clustering and Visual Analysis.** The rolled-up AMT tag results from above were zeroed to make all values positive and then all values were converted into a z-score value using the minimum value observed in all datasets in the place for all missing values. The *Z*-score was calculated as follows:

$$Z_z = \frac{X - \mu_x}{\sigma_x}$$

where $X$ is the individual value, $\mu_x$ is the mean of the values from that row, and $\sigma_x$ is the standard deviation of the values from that row. These transformed values were then exported into a comma-delimited text file containing the protein reference number, number of unique peptides found in each protein and normalized and rolled-up protein abundance values for all of the identified proteins. This file was imported into MeV (http://www.tm4.org/mev.html). For the overall increase/decrease in abundance heat

map representation of the data, the overall experiment analysis (showing both the cleared lysate data and the pellet data) were clustered using a K-means clustering with 5 clusters with average linkage by magnitude and shape with 500 iterations. The cleared lysate data and the pellet data heat maps were obtained using K-means clustering with 5 clusters and 500 iterations.
